# Sexual Behavior Increases Cell Proliferation in the Rostral Migratory Stream and Promotes the Differentiation of the New Cells into Neurons in the Accessory Olfactory Bulb of Female Rats

**DOI:** 10.3389/fnins.2016.00048

**Published:** 2016-02-26

**Authors:** Rebeca Corona, Socorro Retana-Márquez, Wendy Portillo, Raúl G. Paredes

**Affiliations:** ^1^Instituto de Neurobiología, Universidad Nacional Autónoma de MéxicoMexico, Mexico; ^2^Departamento de Biología de la Reproducción, Universidad Autónoma Metropolitana-IztapalapaMexico, Mexico

**Keywords:** neurogenesis and sexual behavior, cell proliferation, cell survival, paced mating, olfactory bulbs

## Abstract

We have previously demonstrated, that 15 days after female rats pace the sexual interaction, there is an increase in the number of new cells that reach the granular cell layer (GrL) of the accessory olfactory bulb (AOB). The aim of the present study was to evaluate, if the first sexual experience in the female rat increases cell proliferation in the subventricular zone (SVZ) and the rostral migratory stream (RMS). We also tested if this behavior promotes the survival of the new cells that integrate into the main olfactory bulb (MOB) and AOB 45 days after the behavioral test. Sexually, naive female rats were injected with the DNA synthesis marker 5′-bromo-2′-deoxyuridine (BrdU) on the day of the behavioral test. They were randomly divided into the following groups: Female rats placed alone in the mating cage (1); Females exposed to amyl acetate odor [banana scent, (2)]; Females that could see, hear, and smell the male but physical contact was not possible [exposed to male, (3)]; Female rats that could pace the sexual interaction (4); and females that mated without the possibility of pacing the sexual interaction (5). Animals were sacrificed 2 days after the behavioral test (proliferation) or 45 days later (survival). Our results show that 2 days after females were exposed to banana scent or to the male, they had a higher number of cells in the SVZ. Females, that mated in pace and no-paced conditions had more new cells in the RMS. At 45 days, no significant differences were found in the number of new cells that survived in the MOB or in the AOB. However, mating increased the percentage of new cells, that differentiated into neurons in the GrL of the AOB. These new cells expressed c-Fos after a second sexual encounter just before the females were sacrificed. No significant differences in plasma levels of estradiol and progesterone were observed between groups. Our results indicate that the first sexual experience increases cell proliferation in the RMS and mating 45 days later enhances the number of new cells that differentiate into neurons in the AOB. These new neurons are activated by sexual stimulation.

## Introduction

Paced mating increases the probability of reproductive success and augments, the rewarding properties of mating. In pacing behavior, the female controls the sexual interaction by regulating the frequency and the temporal pattern of the stimuli received from the male, favoring long periods between each intromission, making the sexual interaction less intense and more efficient (Erskine, 1995; Paredes and Vazquez, 1999; Sugai et al., 2006; Corona and Paredes, 2012). Pacing promotes the acute secretion of luteinizing hormone (LH), prolactin (PRL), and extracellular dopamine in the nucleus accumbens and striatum (Erskine and Hanrahan, 1997). The vaginocervical stimulation received in paced mating increases PRL secretion raising the percentage of pregnant females in comparison to females that did not pace the sexual interaction (McClintock and Adler, 1978; McClintock and Anisko, 1982; Sugai et al., 2006). In female rats, olfactory cues are involved in mate selection as well as in the display and maintenance of the sexual interaction. There are two anatomically separated areas within the OB that process these sexually relevant cues: the main (MOB) and the accessory olfactory bulb (AOB) (Portillo and Paredes, 2004; Bagley et al., 2007). Both the MOB and the AOB continuously add new cells (interneurons) throughout life. Addition of the new interneurons (periglomerular and granular cells) is important for the normal function of the OB since they regulate the activity of projection neurons (mitral cells). Mitral cells process and send the olfactory signal to the rest of the olfactory circuit to control social and reproductive behaviors (Brennan, 2004). The new OB cells proliferate mainly in the lateral walls of the ventricles, the subventricular zone (SVZ), but also in the rostral migratory stream (RMS) (Alvarez-Buylla et al., 2002; Gritti et al., 2002). After the new cells are born, they migrate tangentially for about 2 weeks until reaching the OB and incorporating into their final location in the MOB and AOB. Regulation of the neurogenesis process is highly sensitive to internal and external changes, and since the OB is involved in controlling reproductive behaviors in most mammals, the relevance of the neurogenesis process in OB mediated behaviors is well documented (see Oboti et al., 2009; Nunez-Parra et al., 2011; Peretto et al., 2014). For example, in male hamsters the majority of new cells that incorporate into the MOB and AOB are activated in response to mating and a low percentage of them respond to estrous female odors or to the female presence without physical contact (Huang and Bittman, 2002), suggesting a possible role of the new cells in the processing of sexual related odors and sexual behavior. In female mice, where recognition of the sexual partner is essential for the display of sexual behavior and maintenance of pregnancy, the simple exposure to male pheromones increases proliferation in the SVZ and the number of new cells in the MOB (Mak et al., 2007), and in the AOB (Takahashi et al., 2009). It is well documented that in mice a recently mated female exposed to chemosignals from an unfamiliar male will undergo pregnancy block and will return to estrous, a phenomenon known as the Bruce effect (Bruce, 1959). In contrast, if the female is exposed to the same male she mated with, pregnancy will continue. Females treated with cytosine arabinose (Ara-C), an antimitotic drug that inhibits cell proliferation, show a high rate of pregnancy failure as if the treatment switched the effect from familiar odor to an unfamiliar one (Oboti et al., 2011), further suggesting that the new cells that reach the OB have an important role in reproductive behaviors. The changes during the reproductive cycle seem to be related to OB neurogenesis, since the induction of estrous promotes an increase in proliferation in the SVZ in the female prairie vole (Smith et al., 2001) and in the RMS of female rats (Díaz et al., 2009).

Results from our research group have shown that sexual behavior *per se* could also regulate OB neurogenesis. Sexually experienced male rats injected with the DNA synthesis marker 5′-Bromo-2′-deoxyuridine (BrdU) and allowed to copulate the same day, showed 15 days later an increase in the number of new cells in the granular cell layer (GrL) of the AOB only when males regulate the pattern of copulation and ejaculated one or 3 times (Portillo et al., 2012). In female rats, one paced sexual encounter significantly increased the incorporation of new cells into the GrL of the AOB 15 days after mating (Corona et al., 2011). If the stimulus is repeated, that is, if the females experienced additional paced mating once weekly for 3 consecutive weeks the number of new cells incorporated into the GrL of the AOB is further increased. Moreover, these females also showed a higher incorporation of new cells into the MOB (Arzate et al., 2013). Together, these findings suggest, that paced mating promotes clear changes in OB neurogenesis in a short time interval (15 days). However, it is not known if the presence of these new cells in the AOB could result from increased cell proliferation in the SVZ and RMS. We also need to determine if the increase in the new cells is maintained after 15 days and if the new cells actually survive and integrate into the OB circuits. In order to address these possibilities we evaluated cell proliferation in the SVZ and RMS (two days after mating) and cell survival in the OB 45 days after the first sexual encounter in female rats. We also tested the participation of the new cells in sexual behavior by evaluating the immediate early gene expression (c-Fos) after a sexual interaction. We hypothesized that cell proliferation and survival would be increased in those female rats that paced the sexual interaction, and that the new cells that survived would be activated by sexual behavior.

## Materials and methods

To examine the effects of sexual behavior on cell proliferation in the SVZ and RMS and on cell survival in the MOB and AOB we compared females that were allowed to pace (paced) and females that could not pace (non-paced) the sexual interaction. We also included two olfactory stimuli: females that were exposed to a sexually experienced male (without physical contact) and females exposed to amyl acetate (banana scent). An additional group of females was placed alone in the mating cage.

### Animals

Seventy female rats (Wistar), bred in a local colony at the Instituto de Neurobiologia, Universidad Nacional Autónoma de México were used. They weighed between 230–270 g and were bilaterally ovariectomized (ovx) under deep anesthesia (30% xylazine—70% ketamine; Procin, PiSA A099884, and Cheminova KTCHH11L05, respectively). They were allowed to recover from surgery for 1 week before the experiments began. Forty sexually experienced male rats (300–350 g) of the same strain were used for the behavioral tests. Females were housed in a room without males, with controlled temperature (25 ± 1°C) and humidity and under a reverse light-dark cycle (12 h:12 h). They received tap water and food *ad libitum* (Lab Diet Feed PMI, USA). All behavioral tests were performed during the dark phase of the light-dark cycle, under red dim illumination. Experiments were carried out in accordance with the “Reglamento de la Ley General de Salud en Materia de Investigación para la Salud” of the Mexican Health Ministry that follows NIH guidelines for the use and care of animals and approved by the Instituto de Neurobiologia animal care committee.

### Hormonal treatment

**Figure 1:**
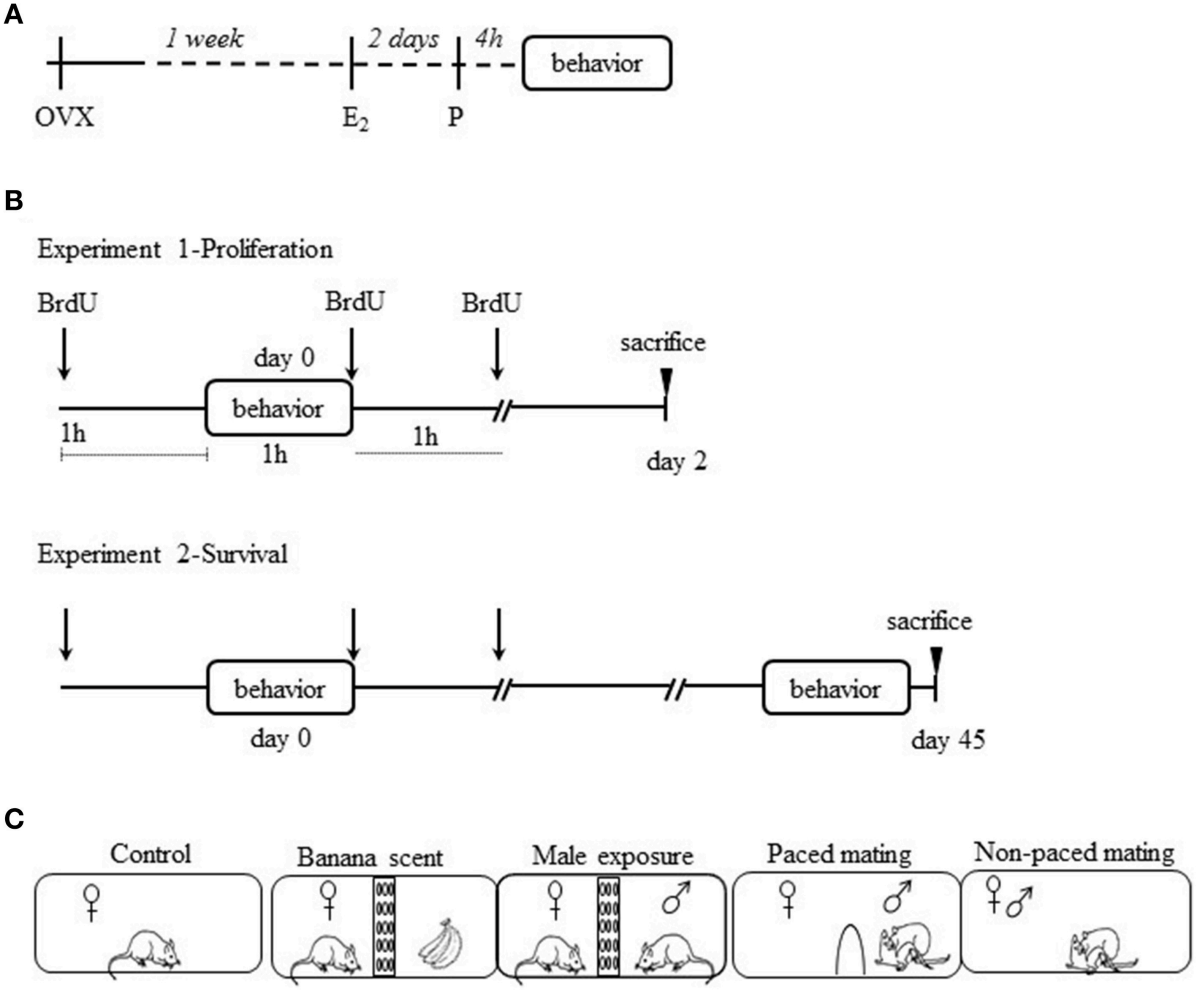
**Experimental Procedure. (A)** ovariectomized (ovx) females were treated with estradiol benzoate (E_2_) and progesterone (P) to induce sexual receptivity. **(B)** Ovx females were injected with 5′-bromo-2′-deoxyuridine (BrdU) three times: 1 h before the behavioral test, immediately after the test and 1 h after the test. They were sacrificed 2 days later for the proliferation experiment and the other groups were sacrificed 45 days later for the survival experiment. **(C)** Schematic representation of the experimental groups.

To induce sexual receptivity in the experimental females, they were subcutaneously injected (sc) with estradiol benzoate (EB, 25 μg/rat; Sigma Aldrich, E-8515) 48 h and with progesterone (P, 1 mg/rat; Sigma Aldrich, P0130-25G) 4 h before the behavioral test (Figure 1A).

### Hormonal levels measurement

In experiment 2, 45 days after BrdU administration ovx females were treated with EB and P and tested according to their corresponding group. At the end of testing, they were anesthetized whit pentoparbital and the blood samples were obtained by cardiac puncture. Plasma levels of estrogen (E2) and progesterone (P) were determined using ELISA (enzyme-linked immunosorbent assay; Diagnostica Internacional Foster City, California, USA). We followed the procedure previously reported (Arzate et al., 2013). Briefly, 25 μl samples of standards or plasma from control and experimental animals were placed in a 96-well microtiter plates; hormone-horseradish per-oxidase conjugated to E2 or P and either rabbit anti-E2 or anti-P, respectively were added and incubated. The microwells were rinsed and incubated with 3,3′,5,5′-tetramethylbenzidine reagent and finally with 1N HCl to stop the reaction. The absorbance was read at 450 nm in an automatic ELISA Microplate Reader (Bio-Rad Hercules, CA, USA). The inter-assay and intra-assay coefficients of variation were: for E2 18.82 and 9.35%, and for P 16.02 and 7.91%, respectively (Tietz, 1995; Pettit et al., 2012).

### Experimental procedure

In order to evaluate the effect of female sexual behavior on OB neurogenesis, we designed two experiments. In experiment 1 (cell proliferation) 35 females of the different groups were sacrificed 2 days after the behavioral tests and cellular proliferation was evaluated in the SVZ and the RMS (Figure 1B). In experiment 2 (cell survival) 35 females of the different groups were sacrificed 45 days after the behavioral tests (Figure 1B). Cell survival, percentage of new cells that differentiated into neurons, and new cells activated after mating (as evaluated by Fos expression) were quantified in the AOB and MOB. The groups and behavioral tests were identical in both experiments. The behavioral tests lasted 1 h and were performed in odorless acrylic cages (62 × 29 × 42 cm) with fresh sawdust covering the floor. Sexually naive females were randomly distributed in the following groups (*n* = 7 per group): (1) Control, females were gently placed in the mating cages, without sexual or olfactory stimulation; (2) Females exposed to amyl acetate (banana scent), subjects were placed in a cage divided in two equal compartments by an acrylic screen with small holes (1 cm diameter). A container fixed to the wall of the screen contained a filter paper moistened with 10 μl of a 10% solution of amyl acetate (Sigma Aldrich, W50400-9); (3) Exposed to male, females were placed in a cage as for group 2, but instead of the container with the banana scent a sexually experienced male was placed in the opposite compartment. In this way females were able to hear, see and smell the active male, but no physical contact was possible; (4) Paced mating, females in this group were allowed to mate pacing the sexual interaction. The tests were performed in the mating cage divided in two equal compartments by an acrylic screen. In the bottom of the screen there was a hole (7 × 4 cm), that allowed the female, but not the male, to go back and forth from the compartment where the male was placed. The hole was too small for the male to go through and in this way the female controlled the rate of the sexual stimulation; (5) Non-paced mating, females in this group mated with a sexually active male without the barrier; in this condition females were not able to pace the sexual interaction (Figure 1C). For experiment 1, females were left in their home cage for the next 2 days. For experiment 2 females were housed with other females of the same group (3 or 4 females per cage) for 45 days. On day 45 they were tested 1 h for sexual behavior with the same male they had mated before and 30 min later they were sacrificed in order to evaluate the activation of the new cells after mating. Females were placed in a separate room with reduce noise, they were gently anesthetized to minimize unspecific c-fos activation.

### Behavioral measures

The lordosis quotient (LQ) was used as a measure of sexual receptivity, and was calculated as the total number of lordosis responses divided by the number of mounts multiplied by 100. Lordosis responses were scored from 0 to 2 as follows: 0 no lordosis displayed; 1 Slight flexion of the back, 2 flexion of the back, head and tail. Lordosis intensity (LI) was calculated as the sum of lordosis score divided by the number of mounts received from the male (Hardy and DeBold, 1972). The number and latencies of mounts, intromissions and ejaculations, and the inter-intromission interval (III) were also recorded. In the paced mating group, the percentage of exits after a mount, intromission, and ejaculation and the return latencies after a mount, intromission, and ejaculation were also recorded.

### Cell proliferation and survival detection by BrdU (5′-bromo-2′deoxyuridine)

BrdU is broadly used to label new born cells, and is an indicator of DNA synthesis (Shingo et al., 2003; Ming and Song, 2005, 2011). BrdU (Sigma Aldrich, B5002-5G) was administrated three times (each 100 mg/kg in 0.9% NaCl for a total dose of 300 mg/kg): 1 h before the test, immediately after the behavioral test and 1 h after the test (Figure 1B).

### Brain-tissue preparation

At the end of each experiment, proliferation (2 days) and survival (45 days) the brain tissue was obtained. All the females received a lethal dose of sodium pentobarbital (100 mg/kg, Cheminova). They were intracardially perfused with 0.1 M phosphate-buffered solution (PBS, pH 7.4) followed by 4% paraformaldehyde (Sigma Aldrich, P-6148) in 0.1 M PBS. The brains were removed and post-fixed in 4% paraformaldehyde for 1 h before they were placed in 30% sucrose for cryoprotection. The right hemisphere of all the subjects was sliced in the sagittal plane at 30 μm intervals using a microtome (Leica, SM2000R) obtaining serial sections. Only this hemisphere was used because there are no significant differences in cell proliferation in the SVZ, RMS and RMS-OB, between the two brain hemispheres (Díaz et al., 2009). Five sections containing the SVZ and RMS were selected for the proliferation experiment and five slices with the AOB and MOB for the survival experiment and processed for immunohistochemical labeling. To analyse cell proliferation we used BrdU-labeling, and BrdU/DCX labeling to identify the cells committed to the neuronal phenotype. BrdU/NeuN labeling was used to identify the mature neuronal phenotype of the new cells, and BrdU/cFos to evaluate the activation of the new cells.

### Immunohistochemistry

#### Protocol for BrdU labeling with peroxidase

Brain slices were processed for floating immunohistochemistry as previously reported (Corona et al., 2011; Portillo et al., 2012; Arzate et al., 2013). Briefly, tissue samples were repeatedly washed in buffer phosphates (Tris hydrochloride, Tris base, and Sodium chloride; J.T Baker) and incubated in 0.5% sodium borohydride (Sigma Aldrich, 452882). Later, the slices were incubated in 1% Triton X-100 (J. T Baker, X198-07), 1% H_2_O2 (J. T Baker, 2189-01), and 1% DMSO (Dimethyl sulfoxide, J. T Baker, 9224-01) solution. Then, cells were incubated with 2N HCl at 36°C for 1 h (J. T Baker, 5616-02). Non-specific epitopes were blocked with 0.1% bovine albumin (Sigma Aldrich A-91418) and 0.3% Triton X-100. Tissues were incubated with primary antibody against BrdU for 48 h at 4°C then washed and incubated for 2 h at room temperature with the secondary antibody diluted in 0.32% Triton X-100 and 0.1% bovine albumin. Brain sections were then rinsed and incubated in Avidin Biotin Complex (AB elite kit; Vector Laboratories, PK-6100) for 90 min at room temperature. Brain sections were rinsed and revealed with the chromogen solution nickel chloride-3,3′-diaminobenzidine (DAB; Vector Laboratories, D5637) and H_2_O_2_. Finally, the reaction was stopped by washing the slices in buffer solution. The brain slices were mounted onto gelatin-coated slides and cover slipped using permount (Fisher, SP 15-500; see list of antibodies, Table 1).

**Table 1:** **List of antibodies used in both experiments**.

**Antibody**	**Species**	**Dilution**	**Suuplier**
**PRIMARY ANTIBODIES**			
BrdU	Mice	1:2000	BD bioscience
BrdU	Rat	1:800	AbD serotec
DCX	Guinea pig	1:500	Millipore
NeuN	Mice	1:250	Millipore
cFos	Rabbit	1:250	Santa Cruz Biotech
**SECONDARY ANTIBODIES**			
IgG biotinylated	Mice	1:500	Vector
Alexa 488	Rat	1:1250	Invitrogen
CY2	Guinea pig	1:1000	Jackson immuno
CY3	Mice	1:1000	Jackson immuno
Alexa 488	Rabbit	1:1250	Invitrogen

#### Protocol for double labeling with fluorescence

Bran slices were incubated with one of the primary antibodies (BrdU, DCX, NeuN, cFos) for 48 h at 4°C in albumin (0.1%) and Triton X-100 (0.32%). The tissues were washed in TBS containing 0.02% Triton X-100 and 1 % albumin to remove primary antibodies and then were incubated with both secondary fluorescent antibodies for 2 h (Table 1). After the incubation, the samples were rinsed with Triton X-100 (0.02%) in TBS followed by TBS, the brain slices were then mounted onto slides and covered with aqua poly/mount (Polysciences, Inc, 18606).

### Quantification of the cells

**Figure 2:**
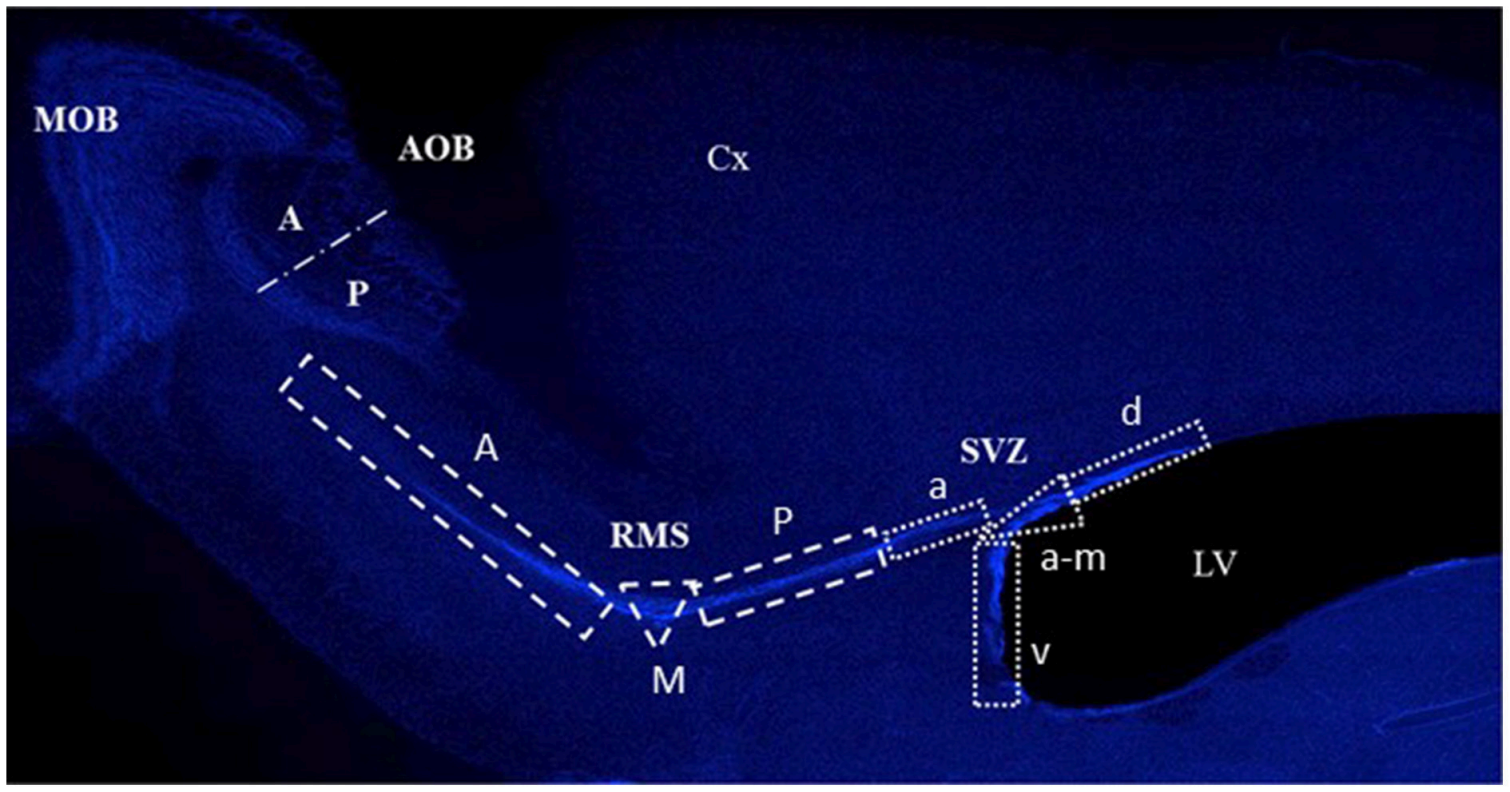
**Photomicrograph showing the different regions where cells were quantified in the subventricular zone (SVZ), the rostral migratory stream (RMS), the main olfactory bulb (MOB), and the accessory olfactory bulb (AOB) in the anterior (A) and posterior (P) region**. The SVZ anterior (A), M (medial) and P (posterior), and in the RMS a (anterior), v (ventral), a-m (anterior-medial), and d (dorsal). The Cortex (Cx) and lateral ventricles (LV) are also indicated.

Images of the five sections of each subject were taken at 10X magnification with a light microscope (Olympus BX60F-3) connected to a motorized slide (Prior ProScan) and the number of BrdU positive cells was counted using the Image Pro software 6.1. During this process, the researcher was blind to the group being analyzed. Reconstructions of these images were used to quantify the BrdU-labeled cells localized in the SVZ, the RMS, the MOB and the AOB (Figure 2).

For experiment 1 (proliferation), the quantification was performed throughout the SVZ and the RMS. Evidence shows that the new OB cells are born in different regions of the SVZ and the RMS (Lois and Alvarez-Buylla, 1993, 1994; Carani et al., 1999; Alonso et al., 2008), therefore we divided the SVZ into dorsal, anterior-medial, ventral, and anterior regions, and the RMS into posterior, medial and anterior regions (Figure 2). Each zone was defined as an area of interest (AOI) with a known area surface and BrdU-labeled cells within its limits were counted. Data are expressed as the number of cells per mm^2^.

**Figure 3:**
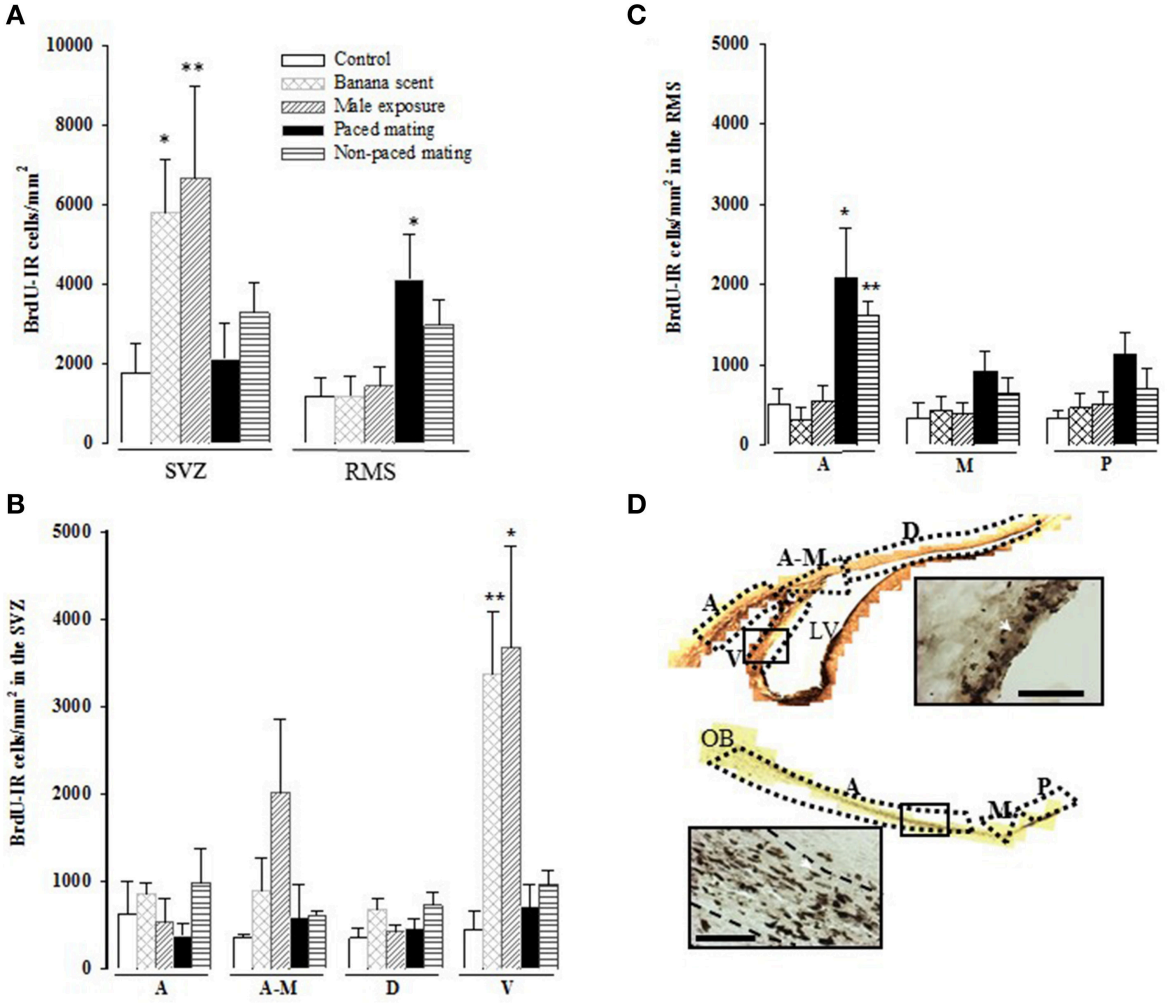
**Cell proliferation in the Subventricular Zone (SVZ) and the Rostral Migratory Stream (RMS) two days after BrdU injection. (A)** BrdU immunoreactive (BrdU-IR) cells in the whole SVZ and RMS; **(B)** BrdU-IR cells in the anterior (A), anterior-medial (A-M), dorsal (D), and ventral (V) sub-regions of the SVZ; **(C)** BrdU positive cells in the anterior (A), medial (M), and posterior (P) sub-regions of the RMS and **(D)** representative photomicrographs of BrdU positive cells in the SVZ and RMS. Arrow heads point to BrdU positive cells. Scale bar = 50 μm. The lateral ventricles (LV) and the olfactory bulb (OB) are also indicated. ^*^Different from control *P* < 0.05, ^**^P < 0.01.

For experiment 2, quantification was performed in the glomerular (GlL) and GrL of the MOB and AOB. For the MOB, three circular AOI's (400 μm diameter) were placed in the apical zone of the GlL and GrL. The AOB, was subdivided into the anterior and posterior regions based on the different functions suggested for them (Martínez-Marcos and Halpern, 1999). Three circular AOI's (200 μm diameter) were placed on the anterior or posterior portion of the GlL and GrL. Five brain sections containing both the MOB and the AOB were counted per subject and seven subjects per group were analyzed. Representative photomicrographs of the BrdU positive cells in the RMS, SVZ and OB are shown in Figures 3D, **5C**.

Double-labeled cells for BrdU/DCX, BrdU/NeuN, and Brdu/cFos were counted using a confocal microscope (Zeiss Inverted LSM 510 and LSM 780) and the image examiner software Aim Image Examiner (Carl Zeiss Microscopy, Jena, Germany). For both experiments, three images were obtained with a 25X objective in each ROI: SVZ (dorsal, anterior-medial, ventral, and anterior), RMS (posterior, medial, and anterior), MOB and AOB (GlL and GrL). For each ROI a z-stack of 12 slices (2 μm per slice) was obtained. Co-localization was verified with the orthogonal tool in the Image Examiner software. Each image was divided into four sections for counting. All the BrdU-labeled cells in each section were counted. We then quantified all the BrdU-labeled cells that colocalized (Figure 6). An average of the four sections was obtained for the three images, and from this data, the percentage of colocalization was calculated as the number of colocalized cells multiplied by 100 and divided by the number of BrdU-labeled cells.

### Statistical analysis

The statistical analysis was performed with SigmaPlot 11.0 software. Since none of the data had a normal distribution, as determined by the Shapiro-Wilk test, non-parametric tests were used. To compare the behavior of paced mating vs. non-paced mating, a Mann-Whitney test was used. The number of cells was analyzed by a Kruskal Wallis test followed by a Mann-Whitney test in case of significant effects. Differences with *p* < 0.05 were considered significant.

## Results

### Experiment 1-proliferation

#### Behavioral measures

##### Paced vs. non-paced mating

All the females were equally receptive at the time of the behavioral test (Table 2). No significant differences were found in the LQ or the LI between paced and non-paced mating (LQ: *U* = 32, *p* = 0.409; LS: *U* = 34, *p* = 0.883). No significant differences were found in the number of mounts (*U* = 22, *p* = 0.536), intromissions (*U* = 16.5, *p* = 0.067), and ejaculations (*U* = 22, *p* = 0.313) or in mount (*U* = 10, *p* = 0.081), intromission (*U* = 23, *p* = 0.229) and ejaculation latencies (*U* = 35, *p* = 0.962). As expected, and as described in previous studies comparing paced and non-paced mating, the inter intromission interval was higher in the pacing group (*U* = 10, *p* = 0.014). As well the percent of exits and return latencies was higher after ejaculation than after intromissions, which in turn was higher than after mounts.

**Table 2:** **Sexual behavior parameters in the proliferation experiment in females that did or did not pace the sexual interaction**.

**Behavioral measures**	**Groups**	
	**Paced mating**	**Non-paced mating**
LQ (%)	99.6±0.44	100
LI	1.86±0.04	1.86±0.05
**NUMBER**		
Mounts	11.1±3	15.7±4.3
Intromissions	30.1±30.7	40±2.8
Ejaculations	2.4±0.24	3±0.4
**LATENCIES (SEC)**		
Mounts	69.4±25.9	276.8±120.1
Intromissions	371.2±211.9	479.9±168.3
Ejaculations	1418.9±330.3	1297.5±164.6
III (sec)	105.1±10.6^*^	64.4±7.7
**PERCENTAGE OF EXITS AFTER**		
Mounts	20.44±6.9	
Intromissions	61.9±4.2	
Ejaculations	100	
**RETURN LATENCIES (SEC) AFTER**		
Mounts	46.6±27.8	
Intromissions	51.7±14.4	
Ejaculations	366.7±79.7	

*LQ, lordosis coefficient; LI, lordosis intensity, III, inter intromission interval*.

* *Different from the non-paced mating group, Mann-Whitney U test (U = 10, p = 0.014)*.

##### Cell proliferation in the SVZ

Females exposed to banana scent (*U* = 17, *p* = 0.032) and those exposed to the male (*U* = 16, *p* = 0.009) showed a significant increase in the total number of new cells in the SVZ in comparison to the control group (Figure 3A). When we analyzed cell proliferation in the different regions of the SVZ (anterior, anterior-medial, dorsal, and ventral), the highest density of new cells was localized in the ventral part (*H* = 13.109, *p* = 0.011), in the groups exposed to the banana scent (*U* = 0, *p* = 0.008), and the male (*U* = 2, *p* = 0.017) in comparison to the control group. No differences were found in the other regions of the SVZ (Figure 3B).

##### Cell proliferation in the RMS

Cell proliferation in the RMS was significantly different between groups (*H* = 11.140, *p* = 0.025). We found, that the group, that paced the sexual interaction showed a higher density of BrdU-immunoreactive (BrdU-IR) cells compared to control females (*U* = 1, *p* = 0.017). Females, that did not pace the sexual interaction showed an increase in the number of BrdU-IR cells that was not statistically significant (*U* = 4, *p* = 0.052; Figure 3A). When the cell density was analyzed in the different regions of the RMS, the differences were located in the anterior region (*H* = 16.634, *p* = 0.002). Subjects from both paced (*U* = 3, *p* = 0.015) and non-paced mating (*U* = 2, *p* = 0.009) groups had a higher density of new cells than control animals. The number of BrdU-IR cells found in the medial and posterior regions was similar for all groups (Figure 3C).

##### Migrating neuroblasts in the SVZ and RMS

**Figure 4:**
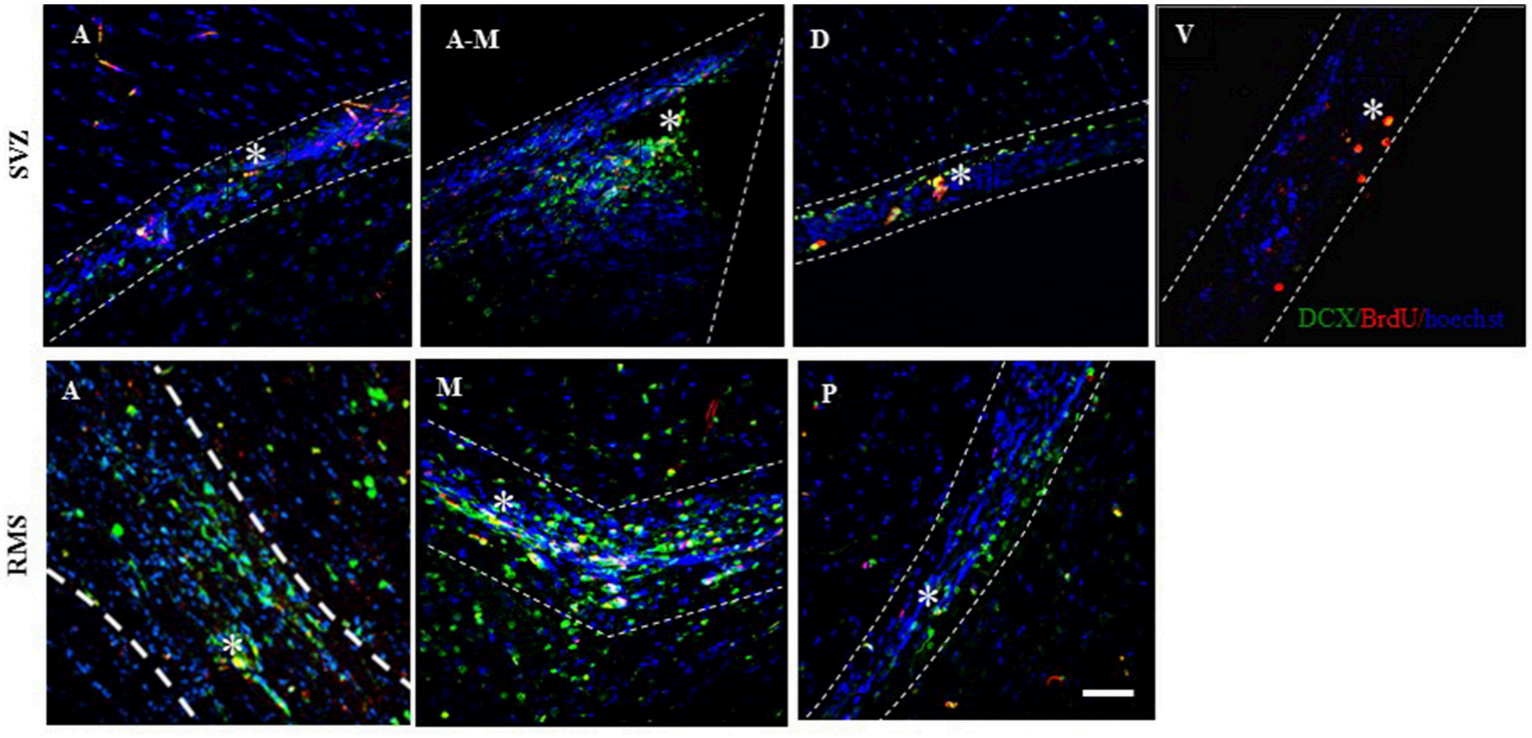
**Representative photomicrographs of the migrating neuroblasts localized in the regions of the Subventricular Zone (SVZ) in the anterior (A), anterior-medial (A-M), dorsal (D), and ventral (V) sub-regions and in the Rostral Migratory Stream (RMS), anterior (A), medial (M), and posterior (P)**. Doublecortin (DCX) positive cells are labeled in green, BrdU positive cells in red, and the cell nuclei in blue (Hoechst). Scale bar = 50 μm. ^*^BrdU/DCX positive cells.

The percentage of new cells (BrdU-IR), that were already committed to the neuronal phenotype and were migrating (DCX-IR) was evaluated in the SVZ and RMS (Figure 4). We found no differences in the total number of the migrating neuroblasts (BrdU-IR/DCX-IR cells) in the SVZ and RMS. When the different regions were analyzed we found, that the groups exposed to banana scent (*U* = 0, *p* = 0.029) and to the male (*H* = 9.947, *p* = 0.041) both had a higher percentage of migrating neuroblasts than the control group in the anterior region of the SVZ (Table 3). No differences were found in the other regions of the SVZ. In the posterior, RMS the group exposed to banana scent had a higher percentage of neuroblasts (*H* = 11.679, *p* = 0.020) than the control group. No differences were found in other areas of the RMS.

**Table 3:** **Percentage of migrating neuroblasts in the Subventricular Zone (SVZ) and in the Rostral Migratory Stream (RMS)**.

**Percentage of BrdU-IR/DCX-IR cells**						
**Regions**		**Groups**				
		**Control**	**Banana scent**	**Male exposure**	**Paced mating**	**Non-paced mating**
SVZ (complete)		35.9±3.8	67.7±3.4	67.5±4.1	53.5±8.1	58.3±14.3
SVZ	Anterior	35.9±6.8	78.8±7.3^*^	73.7±5.1^*^	52.1±5.2	60.8±15.4
	Anterior-Medial	50.5±9.4	65.8±5.5	70.8±10.5	80.6±10	52.8±13.7
	Dorsal	25±16	69.2±7.1	64.3±14.5	58.3±14.4	66.7±15.6
	Ventral	32.4±6.1	57.2±13.6	61.3±5.2	43.1±6.4	70.5±5.8
RMS (complete)		44.9±7.3	62.4±2.3	50.9±5.7	47.5±4.6	60.6±3.4
RMS	Anterior	36.6±15.5	59.7±7.5	63.8±21.9	68±12.0	71±4.2
	Medial	43.7±8.0	54.6±6.7	56.8±2.4	35.5±3.5	54.8±8.1
	Posterior	54.4±8.5	72.9±7.1^*^	32.1±6.6	39.6±6.3	56.2±5.4

* *p < 0.05 different from Control, Mann-Whitney U test*.

### Experiment 2-survival

#### Behavioral measures

##### Paced vs. non-paced mating

To evaluate the effect of sexual behavior on the survival of the new cells in the OB, and the activation of these new cells after sexual behavior, females mated twice with the same male. The first mating test was done on day 0, when BrdU was administered and the second test was 45 days later. No significant differences were found in the LQ and the LI between the paced and non-paced mating groups in either test (Table 4). There was a significant reduction in the number of mounts in test 2 in the females that paced the sexual interaction (*U* = 9.5, *p* = 0.029) in comparison to the females that didn't pace the sexual interaction in the same test. The III was higher in the groups that paced the sexual interaction than in those that didn't. Although these differences were not significant (test 1 *U* = 24, *p* = 0.442; test 2 *U* = 14.5, *p* = 0.065) the change is on the same direction as seen in previous studies (Corona et al., 2011). As observed in experiment 1 the percent of exits and return latencies were higher after ejaculation than after intromissions, which in turn were higher than after mounts.

**Table 4:** **Sexual behavior parameters in the survival experiment in females that paced and in those not allowed to pace the sexual interaction in test 1, when BrdU was administered, and 45 days latter**.

**Behavioral measures**	**Groups**			
	**Pacedmating**		**Non-pacedmating**	
	**Test 1 day 0**	**Test 2 day 45**	**Test 1 day 0**	**Test 2 day 45**
LQ (%)	99.02±0.65	100	100	100
LI	1.84±0.04	1.85±0.07	1.84±0.04	1.85±0.05
**NUMBER**				
Mounts	9.1±2.5	7.6±1.9^*^	21.9±6.5	27.5±6.5
Intromissions	26±5.1	24.6±6.3	34±3.9	32.5±6.4
Ejaculations	3±0.3	3.3±0.6	3.4±0.5	4±0.4
**LATENCIES (SEC)**				
Mounts	88.5±40.6	556.2±372.8	21.8±6.5	27.5±6.0
Intromissions	459.8±208.2	369.1±196.1	34±3.8	32.5±6.4
Ejaculations	1092.9±210.4	1182.6±189.5	1382.2±292.1	901.4±203.2
III (sec)	127.7±24	161.9±28	94.1±5.4	92.5±8.7
**PERCENTAGE OF EXITS AFTER**				
Mounts	42.75±9.9	39.5±11.2		
Intromissions	57.38±7.5	78.25±7.8		
Ejaculations	98.88±3.12	100		
**RETURN LATENCIES (SEC) AFTER**				
Mounts	50.8±25.4	56.2±39.4		
Intromissions	72.6±21.2	57±11.6		
Ejaculations	164.9±50.4	200.1±61.6		

*LQ, lordosis coefficient; LI, lordosis intensity, III, inter intromission interval*.

* *Different from non-paced mating group on test 2, p < 0.05*.

##### Hormonal levels

The plasma levels of E2 and P were quantified in order to exclude the possibility, that the effects on new cell survival were due to hormonal differences. The plasma levels of E2 and P were similar in all the groups (E2: *F* = 1.033, *p* = 0.419; P: *F* = 0.930, *p* = 0.467). The plasma levels (expressed as pg/ml mean ± sem) for E2 were: control 38.8 ± 12, odor exposure 25.1 ± 7.5, exposed to male 33.4 ± 2.5, paced mating 40 ± 3.5, non-paced mating 35.9 ± 4.7. For P: control 15.4 ± 6.4, odor exposure 25.2 ± 2.7, exposed to male 15 ± 4.4, paced mating 17.1 ± 4.1, non-paced mating 21.6 ± 5.2.

##### Cell survival in the OB

**Figure 5:**
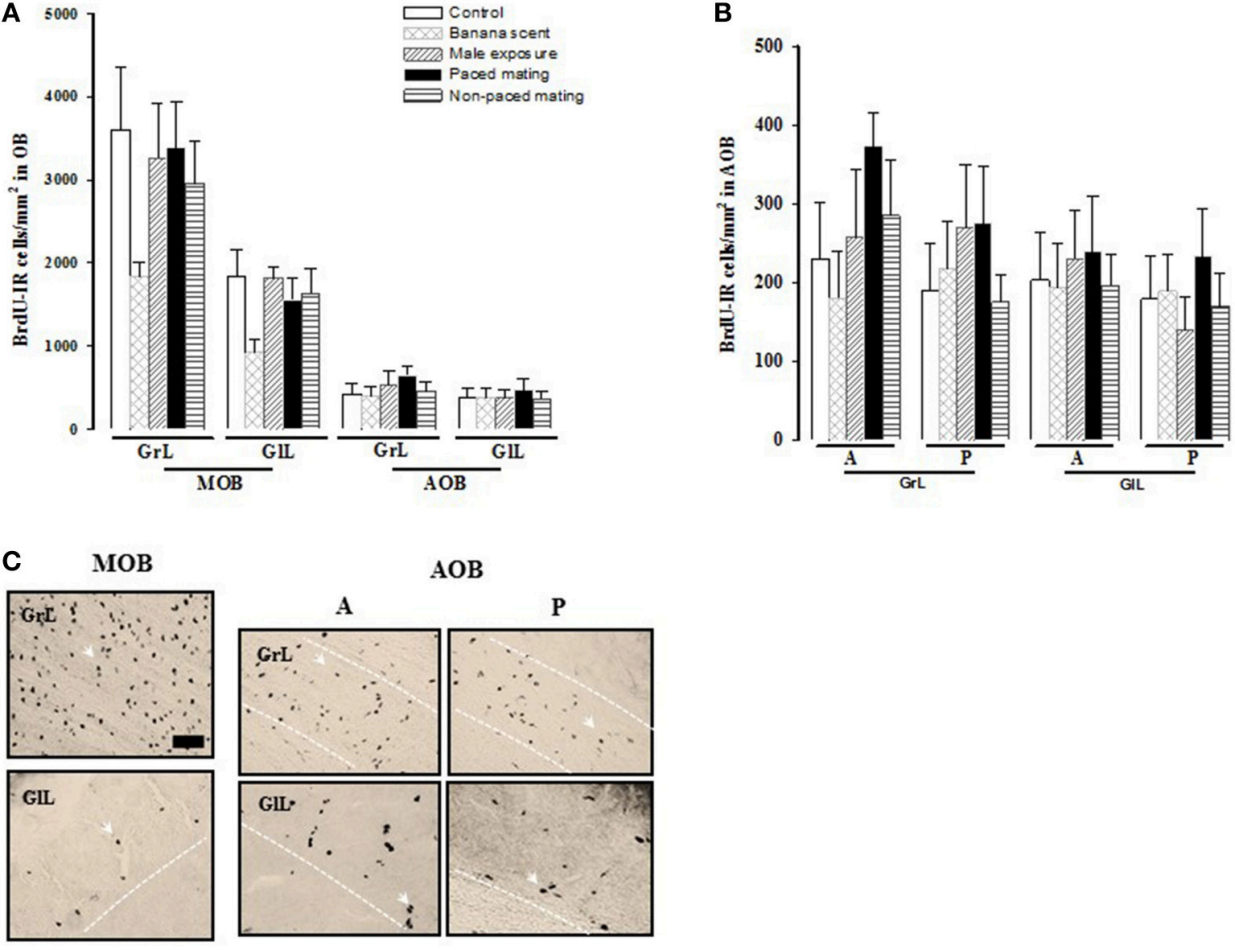
**Number of new cells that survive (A) in the granular (GrL) and glomerular cell layer (GlL) of the Main Olfactory Bulb (MOB) and Accesory Olfactory Bulb (AOB) 45 days after BrdU injection. (B)** Number of BrdU positive cells in the anterior (A) and posterior (P) region in the GrL and GlL of the AOB). **(C)** Representative photomicrographs of the BrdU positive cells in the GrL and GlL of the MOB and in the A and P regions of the AOB. Arrow heads point to BrdU positive cells. Scale bar = 50 μm.

No significant differences were found in the number of BrdU-IR cells that survived 45 days after mating in the different layers of the MOB and the AOB (Figure 5A). Similarly, no differences were found between the anterior and posterior regions of the AOB (Figure 5B).

**Figure 6:**
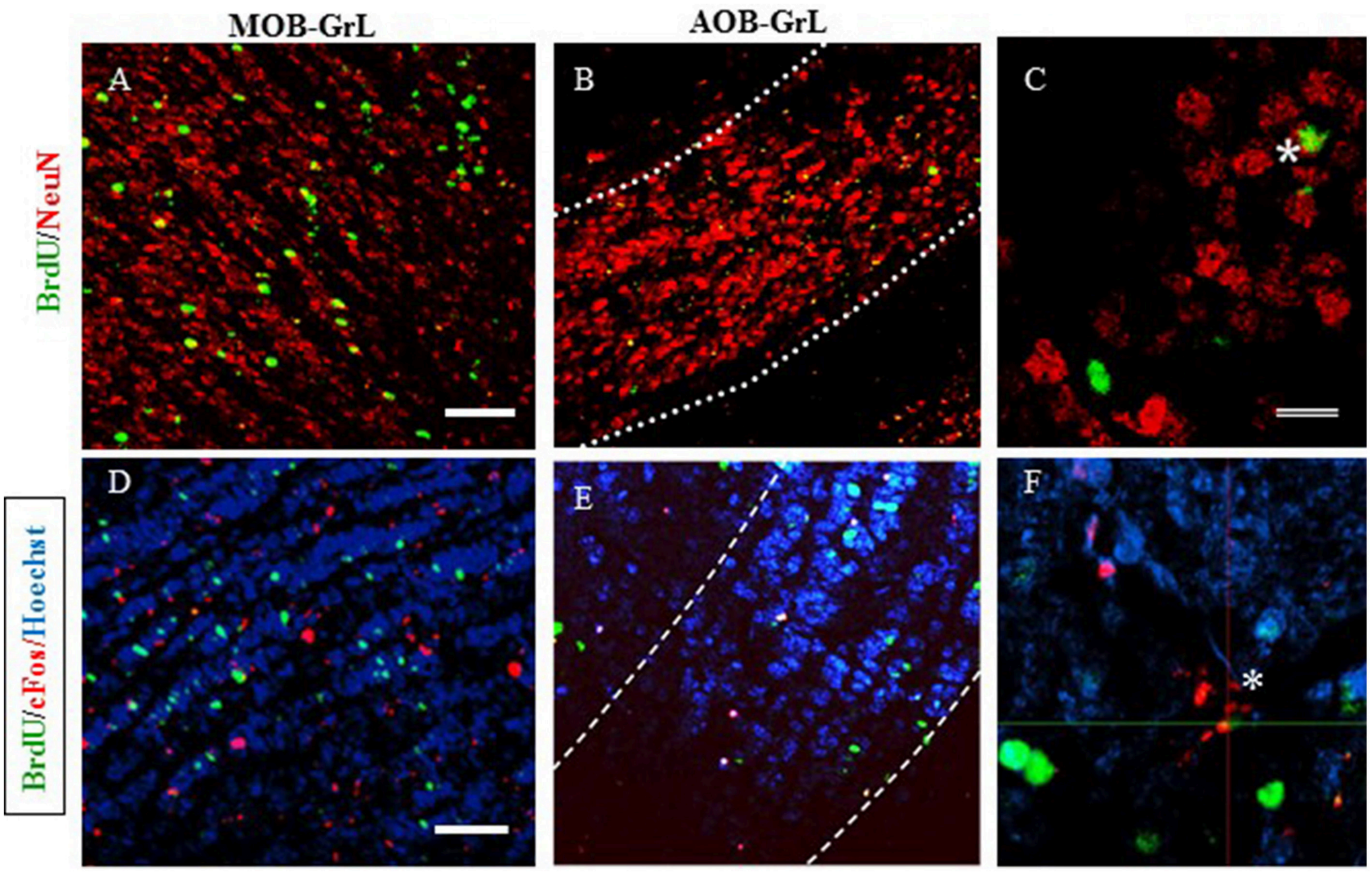
**Representative photomicrographs of new neurons (A–C) in: (A) the granular layer (GrL) of the Main Olfactory Bulb (MOB) and (B) the Accesory Olfactory Bulb (AOB)**. **(C)** High magnification of the BrdU/NeuN positive cells. **(D–F)** new cells that are activated after the second sexual behavior test (BrdU/cFos) in the GrL of the MOB **(D)** and the GrL of the AOB **(E)**. High magnification of a BrdU/cFos positive cell. Scale bar = 50 μm. ^*^Double positive cells for BrdU and NeuN.

##### New neurons in the MOB and AOB

In order to characterize the mature neuronal phenotype of the new cells, the colocalization of BrdU and NeuN markers was counted and the results expressed as the percentage of newborn cells that are neurons in the MOB and AOB (see Table 5A). No differences were found in the different layers of the MOB. In the GrL of the AOB the group that paced the sexual interaction had a higher percentage of new neurons (*U* = 0, *p* = 0.029) than the control group. In the anterior and posterior layers of the AOB no differences were observed in the GlL but in the GrL significant differences were found. Both mating groups had a significantly higher percentages of BrdU-NeuN IR cells (Paced *U* = 0, *p* = 0.029; non-paced *U* = 0, *p* = 0.029) than the control group. The group exposed to the male had significantly lower percentage of new neurons (*U* = 0, *p* = 0.029) in comparison to the control group. No changes were observed in the percentage of new neurons in the AOB or the MOB in the animals expose to banana scent.

**Table 5:** **Percentage of new neurons (A) and percentage of new cells activated (B) after the second mating test in the different groups in the MOB and AOB**.

**A. Percentage of BrdU-IR/NeuN-IR cells**						
**Regions**		**Groups**				
		**Control**	**Banana scent**	**Male exposure**	**Paced mating**	**Non-paced mating**
MOB	GrL	78.71±4.8	81.7±8.5	69.3±7.5	85.2±2.3	79.2±4.5
	GlL	33.3±11.8	25±14.4	58.9±22.3	35.4±14.6	37.5±23.9
AOB	GrL	19.6±1.5	16.3±2.6	11.6±1.2	46.1±11.1^*^	26.5±5
	GlL	25±14.4	76.3±10.3	37.1±10.3	27.1±10.4	18.8±12
	GrL					
	*A*	19.8±1.3	14.8±2.3	12.5±1.2^*^	37.8±8.2^*^	35.5±9.5^*^
	*P*	19.5±2.7	17.8±4.2	10.8±3.5	55±19.3	18±3
	GlL					
	*A*	13±12.5	90±10	28±11.1	59.5±21.2	37.5±23.9
	*P*	50±28.8	53±21	54.3±20.6	54.2±20.8	37.5±23.9
**B. Percentage of BrdU-IR/cFos-IR cells**						
MOB	GrL	32.2±12.3	51.4±3.3	28±10	47.1±5	32.2±9
	GlL	25.3±11.8	40±17	32.6±6.3	60±16.3	60.4±7.5
AOB	GrL	35.1±5.4	3.1±8.5	36.2±3.7	65.1±8^*^	49.4±7.5
	GlL	18.1±7.2	36.5±2.1	27±7	49.2±13	59.1±5.6^*^
	GrL					
	*A*	26.8±7.5	28±10	47.1±5	64.3±4.2^*^	59±15
	*P*	43.3±3.6	32.2±9	25.3±11.8	66±12	40.1±10
	GlL					
	*A*	12±8	27.4±7.1	23±11.1	68±15^*^	54±5^*^
	*P*	24.4±12	46±8.0	31.5±7.7	31±12.2	65±8.6

* *Different from the control group in the same layer, p < 0.05*.

##### New cell activation in the MOB and AOB

The new cells (BrdU-IR) that were also activated, as evaluated by cFos-IR, were quantified and the results are reported as percentage of BrdU-IR/cFos-IR (Table 5B). No differences were found in the percentage of new cells activated in the MOB. Significant differences were found in the AOB. In the GrL, the paced mating group had a significantly higher percentage of activated new neurons than the control group (*U* = 0, *p* = 0.029). In the GlL, the non-paced mating group had the highest percentage of new neurons that were activated (*U* = 0, *p* = 0.029). When the anterior and posterior regions of the AOB were analyzed, the paced mating group had a significantly higher number of activated new neurons in the anterior region of the GrL (*U* = 0, *p* = 0.029) and GlL (*U* = 0, *p* = 0.029) than the control group. In the anterior GlL the non-paced group also showed a higher number of activated neurons than the control group (*U* = 0, *p* = 0.029). In the banana scent group, no changes were observed in the percentage of BrdU-/cFos- IR cells in the AOB and MOB.

## Discussion

### Cell proliferation

In the present study, we hypothesized that paced sexual behavior would increase cell proliferation in the SVZ and RMS of female rats. However, in the SVZ a significant increase in cell proliferation was observed only in the groups exposed to amyl acetate (a non-sexual odor) and in the group exposed to the male (without physical contact), suggesting that olfactory stimulation favors the proliferation of new cells in the SVZ. This cell proliferation was higher specifically in the ventral part of the SVZ. In the RMS females displaying sexual behavior either paced or not, showed a higher density of new cells. This suggests that sexual behavior is important for cell proliferation in females independently of the possibility of pacing the sexual interaction. Sexual behavior enhances the number of cells in the anterior region of the RMS. In this region, the number of BrdU-labeled cells was about three times higher than, that found in the other groups. Together, the results of the proliferation study indicate that odors induce cell proliferation in the SVZ and sexual behavior increases cell proliferation in the anterior RMS, independently of whether the female can or cannot pace the sexual interaction.

Cell proliferation in the SVZ and RMS appears to be differentially regulated. It has been shown that pregnancy and mating with vasectomized males increases the production of neural progenitors in the SVZ (Shingo et al., 2003). In sheep, estrous females in contact with an experienced male did not change the rate of proliferation in the SVZ, but parturient females in contact with their lamb showed decreased proliferation in the SVZ (Brus et al., 2010). It has also been shown that the induction of maternal behavior and exposure of nulliparous female rats to pups, increased the number of new cells in the SVZ, independently of pregnancy or lactation (Furuta and Bridges, 2009). When female mice were exposed to odors from male mice for 7 days a significant increase in BrdU labeled cells was observed in the SVZ and dentate gyrus (Mak et al., 2007). This effect appears to be mediated by the MOB because females treated with ZnSO_4_, which eliminates the function of the main olfactory epithelium, didn't show the increase in proliferation (Mak et al., 2007). These observations suggest that cellular proliferation in the SVZ is highly sensitive to sexually and socially relevant olfactory cues. This is consistent with the results of the present study where a significant increase in cell proliferation in the SVZ was observed only in the groups exposed to amyl acetate (a non-sexual odor) or to the male (without physical contact). It is possible that the cellular proliferation in the SVZ could be due to novelty of an olfactory stimulus and/or to the acquisition of a new olfactory memory generated from sexually relevant olfactory cues.

The RMS is primarily recognized as a migratory pathway and its proliferative role is less known (Alonso et al., 2008). In the female prairie vole, cell proliferation in the RMS is promoted by sexually relevant male olfactory cues that induce sexual receptivity (Smith et al., 2001). In addition, the RMS shows differences in cellular proliferation depending on the day of the estrous cycle. When female rats are in estrous, the proliferation rate is increased in the RMS, but not in the SVZ, (Díaz et al., 2009). These changes are not associated with cell death in the RMS but rather they appear to be associated with an increase in the number of new cells (Díaz et al., 2009). Our data indicate that females displaying sexual behavior either paced or not, showed a higher density of new cells in the RMS.

In the present experiment, we characterized the neural phenotype of the new cells using doublecortin (DCX, widely used to identify immature neurons and migrating neuroblast) labeling. In the anterior-SVZ the groups exposed to banana scent and to male odors showed more than 70% of the new cells label as DCX-IR compared to 30% of the control females. In the posterior RMS, a significant increase in the percentage of DCX-IR cells was observed in the group exposed to banana scent compared to the control females. These results suggest, that the olfactory stimuli promote or facilitate neuronal phenotype determination in the new cells in the anterior SVZ and in the posterior RMS. It has been described that each proliferative region of the SVZ and RMS could be regulated by particular information from their microenvironment. The dorsal regions produce periglomerular interneurons that express tyrosine hydroxylase (TH), and the superficial granule cells. The ventral regions generate the periglomerual interneurons that express calbindin-IR, and the deep granular cells; and the anterior region in general produce calretinin expressing periglomerual interneurons, and most of the granular cells (Carani et al., 1999; Merkle et al., 2007). Future studies will need to evaluate the type of cells induced by olfactory stimulus and mating.

#### Cell survival

To evaluate the effect of sexual behavior on cell survival in the MOB and AOB, females were injected with BrdU the same day, that they had the first behavioral test and were sacrificed 45 days later. The survival rate observed in the MOB and AOB was the same for all groups of females, suggesting that one stimulation, either olfactory or mating, was not enough to modify the survival rate of the new cells that reach the OB. We also quantified, cell survival in the anterior and posterior divisions of the AOB, since there is an anatomical, functional, and chemical distinction between them (Bonfanti et al., 1997; Halpern et al., 1998; Martínez-Marcos et al., 2001; Larriva-Sahd, 2008). The anterior division of the AOB has been extensively related to processing of sexually relevant information, and thus an increase in neurogenesis and the activation of the new cells may contribute to process this specific information. The sensory olfactory neurons located in the apical region of the VNO are connected to the anterior division of the AOB, and are activated by male pheromones present in male urine (Inamura et al., 1999a). As well, exposure to male urine induces cFos expression in the anterior division of the AOB (Inamura et al., 1999a,b). When male mice are exposed to urine of female in estrous, increased activation in the anterior division of the GrL and GlL of the BOA is observed (Honda et al., 2008). All of this evidence suggests, that sexually relevant odors activate preferentially cells in the anterior division of the AOB. However, in the present study we did not find differences in the number of new cells between the anterior and posterior divisions of the AOB. We have previously shown that in female rats that paced the sexual interaction once, there is a significant increase in the number of cells that reach the GrL of the AOB 15 days after mating (Corona et al., 2011). If the stimulus is repeated; that is if the females paced the sexual interaction in 3 more sessions a higher number of cells reach the GrL of the AOB. Moreover, a significantly higher number of cells reach the GlL of the AOB and the mitral and GrL of the MOB (Arzate et al., 2013). When the number of neurons was analyzed, a significant increase was observed in the GrL in both, the AOB and MOB suggesting that repeated paced mating promotes the arrival of more new born neurons to the OB (Arzate et al., 2013). Preliminary observations from our group indicate that repeated paced mating also induces a higher number of neurons 45 days after the first mating session. It has been shown that in the process of OB neurogenesis, the new cells have to go through critical steps that will allow them to reach their final destination (Petreanu and Alvarez-Buylla, 2002; Lledo et al., 2006; Ming and Song, 2011). The time that the new cells require to arrive to the OB and incorporate into their respective layers and preexistent circuits is around 45 days in rodents (Petreanu and Alvarez-Buylla, 2002; Ming and Song, 2011). New neurons between 15 and 22 days old show a mature morphology and it has been suggested that the activity of the OB, specifically the mitral and tufted cells, regulates the incorporation of the new cells into the pre-existent circuits, thereby enhancing their survival (Petreanu and Alvarez-Buylla, 2002). During this critical period, 15 days after generation of the new cells, if they don't receive information or are not stimulated the cells will not survive. For example, it has been demonstrated that in anosmic mice with a mutation in a channel of the olfactory sensory neurons of the main olfactory epithelium where odor information does not arrive to the OB a high proportion of the new cells died between days 15 and 45 days after being born, suggesting that constant information from the OB during this critical period is necessary for the survival of the new cells (Petreanu and Alvarez-Buylla, 2002).

### Neuronal phenotype of the new cells

We evaluated the neuronal phenotype of the new cells, that reached the AOB 45 days after mating and found that the percentage of new neurons (BrdU/NeuN-IR cells) in the GrL was higher in the females that paced the sexual interactions (46%) compared with the control females (20%). This increase was specifically observed in the anterior division of the AOB, in which females, that paced and non-paced the sexual interaction showed the highest level of new neurons (38 and 36%, respectively) compared with the control females (20%), and females exposed to males showed the lowest percentage of new neurons (13%). These results suggest, that sexual behavior could influence the neuronal fate of the new OB cells. The percentage of new neurons found in the MOB GlL and GrL was the same for all groups (80 and 30%, respectively). The effect of sexual behavior on the determination of the new AOB cells is exclusively neuronal, since no differences were observed in the percentage of new glial cells (BrdU/GFAP-IR cells; data not shown). We don't have a clear explanation as to why the group exposed to the male showed a significantly lower percentage of new cells in the anterior region of the GrL of the AOB. Since we haven't seen this effect in our previous studies where groups of females have been exposed to male it could be a spurious effect.

### Activation of the new cells in the OB after mating

Once the new OB cells have survived and arrived at their final destination, they become fully integrated into the pre-existent circuits and present synaptic activity and electric responses after stimulation of OB circuits (Carani et al., 1999; Saghatelyan et al., 2003). The new OB cells, in particular the granular cells have unique properties compared to the preexistent granular cells, including an enhanced synaptic plasticity with a higher response to odors, and greater sensitivity to new odors than the preexistent OB granular cells (Magavi et al., 2005; Sinchak et al., 2007; Nissant et al., 2009). In the present study in order to evaluate if the new cells, that reach the OB are activated, females were tested again for sexual behavior before being sacrificed. No differences in the percentage of BrdU/Fos-IR cells were found in the MOB. In the AOB clear differences were observed in the activation of the new cells. Non-paced mating significantly increased the activation of new cells in the GlL, specifically in the anterior region, of the AOB. Paced mating also increased the percentage of BrdU/Fos-IR cells in the GrL, and in the anterior region of the GrL and GlL of the AOB. These results suggest that the new cells that reach the AOB label 45 day before, after a sexual behavior test are preferentially activated by sexual behavior and not by olfactory cues (banana scent) or by sexually relevant olfactory cues. The new cells incorporated into the OB are more sensitive to stimuli between days 28 and 56 after being born because in this period the density of dendritic spines is increased resulting in an intensified production of synaptic contacts; as a result the new cells are more readily activated (Sinchak et al., 2007).

The activation of the new cells in the AOB found in the present study is consistent with the role of the AOB in sexual behavior. Female sexual behavior is initially regulated by the detection of male pheromones by the AOB. Lesions of the vomeronasal organ (VNO), which projects to the AOB, eliminates receptive behavior in hamsters (Mackay-Sim and Rose, 1986), prairie voles (Wysocki et al., 1991), rats (Guarraci and Clark, 2006), and mice (Keller et al., 2006). The AOB receives information directly from several central brain regions like the bed nucleus of the stria terminalis (BNST), rostral and medial portion of the medial amygdala (MeA) and the posteromedial amygdala (Keller et al., 2009). The posteromedial amygdala and the BNST are two of the regions that are activated (measured by Fos expression) after females receive natural or artificial vaginocervical stimulation (VCS; Erskine, 1993; Hosokawa and Chiba, 2005; Sugai et al., 2006). This implies the possibility, that when females receive stimulation from the male during sexual behavior these structures are activated. This activation together with the information that arrives to the AOB could promote the activation of the new neurons. The new neurons in the GrL receive and are activated by centrifugal inputs from the central regions. The periglomerular interneurons, which are also activated after sexual behavior, could be associated with mate discrimination.

Sexual behavior induces a reward state that could enhance the effects observed on OB neurogenesis. Our group has demonstrated that females and males that paced the sexual interaction develop a positive affective, reward, state as evaluated by conditioned place preference (CPP). If the subjects do not pace the sexual interaction they don't develop CPP (Paredes and Alonso, 1997; Martínez and Paredes, 2001; Arzate et al., 2011). The reward state in females can be induced by 10 paced intromissions (Paredes and Alonso, 1997) or by 1 h of paced mating (Arzate et al., 2011). The reward state generated by paced mating is mediated by opioids since the administration of naloxone, an opioid antagonist, blocks the CPP induced by paced mating in males (Agmo and Berenfeld, 1990) and females (Paredes and Martínez, 2001; García-Horsman et al., 2008). Since, opioids are involved in the process of cell death and survival (Tegeder and Geisslinger, 2004) and inhibit neurogenesis in the adult rat hippocampus (Eisch et al., 2000), future studies will need to address if neurogenesis induced by paced mating can be block by opioid antagonists.

The results observed in the present experiment are not due to differences in E2 or P plasma levels. All groups had the same hormonal levels when sacrificed. Similar results were obtained when females were mated repeatedly under pacing conditions. We found a higher number of cells and neurons (see above) but no differences were found in E2 and P levels 15 days after mating (Arzate et al., 2013).

To summarize, one sexual encounter promotes changes in the OB neurogenesis process. Mating increases the proliferation of new cells in the RMS particularly in the anterior region, favors the incorporation of new cells in the GrL of the AOB and promotes commitment to the neuronal lineage of the new cells, increasing the percentage of new neurons in the GrL of the AOB, especially in the anterior division. The new cells, that reach the GrL of the AOB anterior division are preferentially activated by mating. Together, these results strongly suggest, that mating behavior influences the process of OB neurogenesis and that the new cells that incorporate into the AOB have a relevant function in female sexual behavior.

## Author contributions

RC help in the design of the study, performed the experiments and help writing the paper. SR performed the hormonal assay of the study and help writing the paper. WP supervised the behavioral tests, analyzed the data and help writing the paper. RP designed the study, help in data analysis, help writing the paper, and obtained funding.

### Conflict of interest statement

The authors declare that the research was conducted in the absence of any commercial or financial relationships that could be construed as a potential conflict of interest.

## References

[B1] Agmo A. Berenfeld R. (1990). Reinforcing properties of ejaculation in the male rat: role of opioids and dopamine. Behav. Neurosci. 104, 177–182. 10.1037/0735-7044.104.1.177 2156520

[B2] Alonso M. Ortega-Pérez I. Grubb M. S. Bourgeois J. P. Charneau P. Lledo P. M. (2008). Turning astrocytes from the rostral migratory stream into neurons: a role for the olfactory sensory organ. J. Neurosci. 28, 11089–11102. 10.1523/JNEUROSCI.3713-08.2008 18945916 PMC6671355

[B3] Alvarez-Buylla A. Seri B. Doetsch F. (2002). Identification of neural stem cells in the adult vertebrate brain. Brain Res. Bull. 57, 751–758. 10.1016/S0361-9230(01)00770-5 12031271

[B4] Arzate D. M. Portillo W. Corona R. Paredes R. G. (2013). Repeated paced mating promotes the arrival of more newborn neurons in the main and accessory olfactory bulbs of adult female rats. Neuroscience 232, 151–160. 10.1016/j.neuroscience.2012.12.014 23262235

[B5] Arzate D. M. Portillo W. Rodríguez C. Corona R. Paredes R. G. (2011). Extended paced mating tests induces conditioned place preference without affecting sexual arousal. Horm. Behav. 59, 674–680. 10.1016/j.yhbeh.2010.08.016 20816964

[B6] Bagley J. LaRocca G. Jimenez D. A. Urban N. N. (2007). Adult neurogenesis and specific replacement of interneuron subtypes in the mouse main olfactory bulb. BMC Neurosci. 8:92. 10.1186/1471-2202-8-92 17996088 PMC2238759

[B7] Bonfanti L. Peretto P. Merighi A. Fasolo A. (1997). Newly-generated cells from the rostral migratory stream in the accessory olfactory bulb of the adult rat. Neuroscience 81, 489–502. 10.1016/S0306-4522(97)00090-0 9300436

[B8] Brennan P. A. (2004). The nose knows who's who: chemosensory individuality and mate recognition in mice. Horm. Behav. 46, 231–240. 10.1016/j.yhbeh.2004.01.010 15325224

[B9] Bruce H. M. (1959). An exteroceptive block to pregnancy in the mouse. Nature 184, 105. 10.1038/184105a0 13805128

[B10] Brus M. Meurisse M. Franceschini I. Keller M. Lévy F. (2010). Evidence for cell proliferation in the sheep brain and its down-regulation by parturition and interactions with the young. Horm. Behav. 58, 737–746. 10.1016/j.yhbeh.2010.07.006 20692260

[B11] Carani C. Rochira V. Faustini-Fustini M. Balestrieri A. Granata A. R. (1999). Role of oestrogen in male sexual behaviour: insights from the natural model of aromatase deficiency. Clin. Endocrinol. 51, 517–524. 10.1046/j.1365-2265.1999.00849.x 10583321

[B12] Corona R. Larriva-Sahd J. Paredes R. G. (2011). Paced-mating increases the number of adult new born cells in the internal cellular (granular) layer of the accessory olfactory bulb. PLoS ONE 6:e19380. 10.1371/journal.pone.0019380 21637743 PMC3103495

[B13] Corona R. P. W. Paredes R. (2012). Neuronas y Sexo. Mexico, MX: Publicaciones cbs y Universidad Autónoma Metropolitana.

[B14] Díaz D. Valero J. Airado C. Baltanás F. C. Weruaga E. Alonso J. R. (2009). Sexual dimorphic stages affect both proliferation and serotonergic innervation in the adult rostral migratory stream. Exp. Neurol. 216, 357–364. 10.1016/j.expneurol.2008.12.013 19162010

[B15] Eisch A. J. Barrot M. Schad C. A. Self D. W. Nestler E. J. (2000). Opiates inhibit neurogenesis in the adult rat hippocampus. Proc. Natl. Acad. Sci. U.S.A. 97, 7579–7584. 10.1073/pnas.120552597 10840056 PMC16588

[B16] Erskine M. S. (1993). Mating-induced increases in FOS protein in preoptic area and medial amygdala of cycling female rats. Brain Res. Bull. 32, 447–451. 10.1016/0361-9230(93)90289-N 8221135

[B17] Erskine M. S. (1995). Prolactin release after mating and genitosensory stimulation in females. Endocr. Rev. 16, 508–528. 8521792 10.1210/edrv-16-4-508

[B18] Erskine M. S. Hanrahan S. B. (1997). Effects of paced mating on c-fos gene expression in the female rat brain. J. Neuroendocrinol. 9, 903–912. 10.1046/j.1365-2826.1997.00660.x 9468015

[B19] Furuta M. Bridges R. S. (2009). Effects of maternal behavior induction and pup exposure on neurogenesis in adult, virgin female rats. Brain Res. Bull. 80, 408–413. 10.1016/j.brainresbull.2009.08.011 19712726 PMC2764823

[B20] Garcia-Horsman S. P. Agmo A. Paredes R. G. (2008). Infusions of naloxone into the medial preoptic area, ventromedial nucleus of the hypothalamus, and amygdala block conditioned place preference induced by paced mating behavior. Horm. Behav. 54, 709–716. 10.1016/j.yhbeh.2008.07.011 18721808

[B21] Gritti A. Bonfanti L. Doetsch F. Caille I. Alvarez-Buylla A. Lim D. A.. (2002). Multipotent neural stem cells reside into the rostral extension and olfactory bulb of adult rodents. J. Neurosci. 22, 437–445. 11784788 10.1523/JNEUROSCI.22-02-00437.2002 PMC6758684

[B22] Guarraci F. A. Clark A. S. (2006). Ibotenic acid lesions of the medial preoptic area disrupt the expression of partner preference in sexually receptive female rats. Brain Res. 1076, 163–170. 10.1016/j.brainres.2005.12.120 16473334

[B23] Halpern M. Shapiro L. S. Jia C. (1998). Heterogeneity in the accessory olfactory system. Chem. Senses 23, 477–481. 10.1093/chemse/23.4.477 9759536

[B24] Hardy D. F. DeBold J. F. (1972). Effects of coital stimulation upon behavior of the female rat. J. Comp. Physiol. Psychol. 78, 400–408. 10.1037/h0032536 5067071

[B25] Honda N. Sakamoto H. Inamura K. Kashiwayanagi M. (2008). Changes in Fos expression in the accessory olfactory bulb of sexually experienced male rats after exposure to female urinary pheromones. Eur. J. Neurosci. 27, 1980–1988. 10.1111/j.1460-9568.2008.06169.x 18412619

[B26] Hosokawa N. Chiba A. (2005). Effects of sexual experience on conspecific odor preference and estrous odor-induced activation of the vomeronasal projection pathway and the nucleus accumbens in male rats. Brain Res. 1066, 101–108. 10.1016/j.brainres.2005.10.036 16330001

[B27] Huang L. Bittman E. L. (2002). Olfactory bulb cells generated in adult male golden hamsters are specifically activated by exposure to estrous females. Horm. Behav. 41, 343–350. 10.1006/hbeh.2002.1767 11971669

[B28] Inamura K. Kashiwayanagi M. Kurihara K. (1999a). Regionalization of Fos immunostaining in rat accessory olfactory bulb when the vomeronasal organ was exposed to urine. Eur. J. Neurosci. 11, 2254–2260. 10.1046/j.1460-9568.1999.00646.x 10383614

[B29] Inamura K. Matsumoto Y. Kashiwayanagi M. Kurihara K. (1999b). Laminar distribution of pheromone-receptive neurons in rat vomeronasal epithelium. J. Physiol. 517(Pt 3), 731–739. 10.1111/j.1469-7793.1999.0731s.x 10358114 PMC2269374

[B30] Keller M. Baum M. J. Brock O. Brennan P. A. Bakker J. (2009). The main and the accessory olfactory systems interact in the control of mate recognition and sexual behavior. Behav. Brain Res. 200, 268–276. 10.1016/j.bbr.2009.01.020 19374011

[B31] Keller M. Pierman S. Douhard Q. Baum M. J. Bakker J. (2006). The vomeronasal organ is required for the expression of lordosis behaviour, but not sex discrimination in female mice. Eur. J. Neurosci. 23, 521–530. 10.1111/j.1460-9568.2005.04589.x 16420459 PMC2266683

[B32] Larriva-Sahd J. (2008). The accessory olfactory bulb in the adult rat: a cytological study of its cell types, neuropil, neuronal modules, and interactions with the main olfactory system. J. Comp. Neurol. 510, 309–350. 10.1002/cne.21790 18634021

[B33] Lledo P. M. Alonso M. Grubb M. S. (2006). Adult neurogenesis and functional plasticity in neuronal circuits. Nat. Rev. Neurosci. 7, 179–193. 10.1038/nrn1867 16495940

[B34] Lois C. Alvarez-Buylla A. (1993). Proliferating subventricular zone cells in the adult mammalian forebrain can differentiate into neurons and glia. Proc. Natl. Acad. Sci. U.S.A. 90, 2074–2077. 10.1073/pnas.90.5.2074 8446631 PMC46023

[B35] Lois C. Alvarez-Buylla A. (1994). Long-distance neuronal migration in the adult mammalian brain. Science 264, 1145–1148. 10.1126/science.8178174 8178174

[B36] Mackay-Sim A. Rose J. D. (1986). Removal of the vomeronasal organ impairs lordosis in female hamsters: effect is reversed by luteinising hormone-releasing hormone. Neuroendocrinology 42, 489–493. 10.1159/000124492 3517669

[B37] Magavi S. S. Mitchell B. D. Szentirmai O. Carter B. S. Macklis J. D. (2005). Adult-born and preexisting olfactory granule neurons undergo distinct experience-dependent modifications of their olfactory responses *in vivo*. J. Neurosci. 25, 10729–10739. 10.1523/JNEUROSCI.2250-05.2005 16291946 PMC6725839

[B38] Mak G. K. Enwere E. K. Gregg C. Pakarainen T. Poutanen M. Huhtaniemi I.. (2007). Male pheromone-stimulated neurogenesis in the adult female brain: possible role in mating behavior. Nat. Neurosci. 10, 1003–1011. 10.1038/nn1928 17603480

[B39] Martínez I. Paredes R. G. (2001). Only self-paced mating is rewarding in rats of both sexes. Horm. Behav. 40, 510–517. 10.1006/hbeh.2001.1712 11716580

[B40] Martínez-Marcos A. Halpern M. (1999). Differential projections from the anterior and posterior divisions of the accessory olfactory bulb to the medial amygdala in the opossum, Monodelphis domestica. Eur. J. Neurosci. 11, 3789–3799. 10.1046/j.1460-9568.1999.00797.x 10583468

[B41] Martínez-Marcos A. Ubeda-Bañón I. Halpern M. (2001). Cell migration to the anterior and posterior divisions of the granule cell layer of the accessory olfactory bulb of adult opossums. Brain Res. Dev. Brain Res. 127, 95–98. 10.1016/S0165-3806(01)00106-7 11287070

[B42] McClintock M. K. Adler N. T. (1978). The role of the female during copulation in wild domestic Norway rats (*Rattus norvegicus*). Behaviour 67, 67–96. 10.1163/156853978X00260

[B43] McClintock M. K. Anisko J. J. (1982). Group mating among Norway rats I. Sex differences in the pattern and neuroendocrine consequences of copulation. Animal Behav. 30, 12 10.1016/S0003-3472(82)80051-1

[B44] Merkle F. T. Mirzadeh Z. Alvarez-Buylla A. (2007). Mosaic organization of neural stem cells in the adult brain. Science 317, 381–384. 10.1126/science.1144914 17615304

[B45] Ming G. L. Song H. (2005). Adult neurogenesis in the mammalian central nervous system. Annu. Rev. Neurosci. 28, 223–250. 10.1146/annurev.neuro.28.051804.101459 16022595

[B46] Ming G. L. Song H. (2011). Adult neurogenesis in the mammalian brain: significant answers and significant questions. Neuron 70, 687–702. 10.1016/j.neuron.2011.05.001 21609825 PMC3106107

[B47] Nissant A. Bardy C. Katagiri H. Murray K. Lledo P. M. (2009). Adult neurogenesis promotes synaptic plasticity in the olfactory bulb. Nat. Neurosci. 12, 728–730. 10.1038/nn.2298 19412168

[B48] Nunez-Parra A. Pugh V. Araneda R. C. (2011). Regulation of adult neurogenesis by behavior and age in the accessory olfactory bulb. Mol. Cell. Neurosci. 47, 274–285. 10.1016/j.mcn.2011.05.003 21600286 PMC3137699

[B49] Oboti L. Savalli G. Giachino C. De Marchis S. Panzica G. C. Fasolo A.. (2009). Integration and sensory experience-dependent survival of newly-generated neurons in the accessory olfactory bulb of female mice. Eur. J. Neurosci. 29, 679–692. 10.1111/j.1460-9568.2009.06614.x 19200078

[B50] Oboti L. Schellino R. Giachino C. Chamero P. Pyrski M. Leinders-Zufall T.. (2011). Newborn interneurons in the accessory olfactory bulb promote mate recognition in female mice. Front. Neurosci. 5:113. 10.3389/fnins.2011.00113 21994486 PMC3182443

[B51] Paredes R. G. Alonso A. (1997). Sexual behavior regulated (paced) by the female induces conditioned place preference. Behav. Neurosci. 111, 123–128. 10.1037/0735-7044.111.1.123 9109630

[B52] Paredes R. G. Martínez I. (2001). Naloxone blocks place preference conditioning after paced mating in female rats. Behav. Neurosci. 115, 1363–1367. 10.1037/0735-7044.115.6.1363 11770067

[B53] Paredes R. G. Vazquez B. (1999). What do female rats like about sex? Paced mating. Behav. Brain Res. 105, 117–127. 10.1016/S0166-4328(99)00087-X 10553695

[B54] Peretto P. Schellino R. De Marchis S. Fasolo A. (2014). The interplay between reproductive social stimuli and adult olfactory bulb neurogenesis. Neural Plast. 2014:497657. 10.1155/2014/497657 25140258 PMC4130132

[B55] Petreanu L. Alvarez-Buylla A. (2002). Maturation and death of adult-born olfactory bulb granule neurons: role of olfaction. J. Neurosci. 22, 6106–6113. 12122071 10.1523/JNEUROSCI.22-14-06106.2002 PMC6757952

[B56] Pettit A. S. Desroches R. Bennett S. A. (2012). The opiate analgesic buprenorphine decreases proliferation of adult hippocampal neuroblasts and increases survival of their progeny. Neuroscience 200, 211–222. 10.1016/j.neuroscience.2011.10.039 22079577

[B57] Portillo W. Paredes R. G. (2004). Sexual incentive motivation, olfactory preference, and activation of the vomeronasal projection pathway by sexually relevant cues in non-copulating and naive male rats. Horm. Behav. 46, 330–340. 10.1016/j.yhbeh.2004.03.001 15325233

[B58] Portillo W. Unda N. Camacho F. J. Sánchez M. Corona R. Arzate D. M.. (2012). Sexual activity increases the number of newborn cells in the accessory olfactory bulb of male rats. Front. Neuroanat. 6:25. 10.3389/fnana.2012.00025 22783170 PMC3390685

[B59] Saghatelyan A. Carleton A. Lagier S. de Chevigny A. Lledo P. M. (2003). Local neurons play key roles in the mammalian olfactory bulb. J. Physiol. Paris 97, 517–528. 10.1016/j.jphysparis.2004.01.009 15242661

[B60] Shingo T. Gregg C. Enwere E. Fujikawa H. Hassam R. Geary C.. (2003). Pregnancy-stimulated neurogenesis in the adult female forebrain mediated by prolactin. Science 299, 117–120. 10.1126/science.1076647 12511652

[B61] Sinchak K. Dewing P. Cook M. Micevych P. (2007). Release of orphanin FQ/nociceptin in the medial preoptic nucleus and ventromedial nucleus of the hypothalamus facilitates lordosis. Horm. Behav. 51, 406–412. 10.1016/j.yhbeh.2006.12.008 17274997 PMC1865518

[B62] Smith M. T. Pencea V. Wang Z. Luskin M. B. Insel T. R. (2001). Increased number of BrdU-labeled neurons in the rostral migratory stream of the estrous prairie vole. Horm. Behav. 39, 11–21. 10.1006/hbeh.2000.1630 11161879

[B63] Sugai T. Yoshimura H. Kato N. Onoda N. (2006). Component-dependent urine responses in the rat accessory olfactory bulb. Neuroreport 17, 1663–1667. 10.1097/01.wnr.0000239950.14954.59 17047450

[B64] Takahashi T. Zhu Y. Hata T. Shimizu-Okabe C. Suzuki K. Nakahara D. (2009). Intracranial self-stimulation enhances neurogenesis in hippocampus of adult mice and rats. Neuroscience 158, 402–411. 10.1016/j.neuroscience.2008.10.048 19041373

[B65] Tegeder I. Geisslinger G. (2004). Opioids as modulators of cell death and survival–unraveling mechanisms and revealing new indications. Pharmacol. Rev. 56, 351–369. 10.1124/pr.56.3.2 15317908

[B66] Tietz N. W. (ed.). (1995). Clinical/Guide to Laboratory Tests, 3rd Edn. Philadelphia, PA: W.B. Saunders, CO.

[B67] Wysocki C. J. Kruczek M. Wysocki L. M. Lepri J. J. (1991). Activation of reproduction in nulliparous and primiparous voles is blocked by vomeronasal organ removal. Biol. Reprod. 45, 611–616. 10.1095/biolreprod45.4.611 1751636

